# Development and application of a proximal hamstring MRI-based scoring tool in patients undergoing proximal hamstring tendon surgical repair

**DOI:** 10.1016/j.jor.2023.10.008

**Published:** 2023-10-10

**Authors:** Jay R. Ebert, William Breidahl, Sven Klinken, Peter T. Annear

**Affiliations:** a School of Human Sciences (Exercise and Sport Science), University of Western Australia, Crawley, Western Australia, 6009, Australia; b HFRC Rehabilitation Clinic, 117 Stirling Highway, Nedlands, Western Australia, 6009, Australia; c Perth Radiological Clinic, Subiaco, Perth, Western Australia, 6008, Australia; d Perth Orthopaedic & Sports Medicine Centre, West Perth, Western Australia, 6005, Australia; e Perth Orthopaedic and Sports Medicine Research Institute, West Perth, Western Australia, Australia

**Keywords:** Proximal hamstring avulsion, Surgical repair, MRI, Clinical outcomes

## Abstract

**Purpose:**

An MRI-based scoring tool assessing surgical repair after proximal hamstring avulsions may provide benefit in the context of research, while serial post-operative MRI will provide insight of what to expect in the clinical context of early re-injury requiring imaging. This study developed and assessed the reliability of a Proximal Hamstring Objective Magnetic Resonance Imaging Score (PHOMRIS), further assessing MRI-based repair status and its correlation with patient-reported outcome.

**Methods:**

15 patients that underwent proximal hamstring surgical repair underwent MRI and clinical review pre-operatively and at 3-, 6- and 12-months. Clinical scores included the Lower Extremity Functional Scale (LEFS), the Perth Hamstring Assessment Tool (PHAT) and Tegner Activity Scale (TAS). The MRI-based tool assessed the conjoint (semitendinosus & biceps femoris) and semimembranosus insertion components based on bone-tendon healing, signal and retraction. Inter- and intra-observer reliability of the tool was assessed.

**Results:**

Inter-observer reliability indicated a strong correlation for the semimembranosus (rho = 0.827, p < 0.0001) and conjoint (rho = 0.851, p < 0.0001) components. Intra-observer reliability indicated a strong correlation for the semimembranosus (rho = 0.852, p < 0.0001) and conjoint (rho = 0.996, p < 0.0001) components. All clinical scores and the semimembranosus hamstrings component MRI score significantly improved (p < 0.05) over time, though the conjoint component did not (p = 0.219). At 12 months, a higher LEFS was significantly associated with a better semimembranosus MRI score (r = −0.57, p = 0.042), though no other significant correlations (p > 0.05) were observed between clinical and MRI measures.

**Conclusions:**

Excellent reliability was observed for the MRI-based scoring tool, which may prove useful in both a research and clinical setting.

## 1 Introduction

While the ideal treatment for 1–2 hamstring tendon proximal avulsions is somewhat unclear and may be dictated by the individual's clinical presentation, degree of retraction, future patient activity demands and surgeon experiences,^1^ complete 3-tendon ruptures may result in an inability to successfully return to athletic activities, let alone activities of daily living (ADLs). Complete proximal hamstring ruptures are defined as the tearing of semimembranosus, semitendinosus and biceps femoris (i.e. all three hamstring tendons).^2^^,^^3^ Early surgical repair (vs non-operative treatment) in these cases results in higher satisfaction, athletic capacity & functional recovery.^4^^,^^5^

Several systematic reviews and meta analyses have reported post-operative clinical outcomes following these complete avulsions,^4^, ^5^, ^6^, ^7^, ^8^ generally via established patient-reported outcome measures (PROMs), patient satisfaction, return to activity/sport status and objective measures of strength and/or functional hop capacity. While a consensus is lacking on the best outcome measures to assess post-operative recovery after surgical repair, a recently published review recommended an increased commitment to PROMs such as the Perth Hamstring Assessment Tool (PHAT) and the Lower Extremity Functional Scale (LEFS), as well as return to sport status and isokinetic strength assessment.^9^

Subsequent to clinical status, magnetic resonance imaging (MRI) has been suggested as the best modality to evaluate these proximal hamstring injuries.^1^ However, while MRI-based classification systems have been reported to diagnose proximal hamstring avulsions,^3^ based on factors such as location, degree of retraction and the appearance of sciatic nerve tethering, little is known about the radiological recovery and/or status of the repair. Of the limited studies that have evaluated the post-operative repair on MRI, Mica et al.^10^ retrospectively reviewed MRIs in six patients at 10–118 months post-surgery, while Chahal et al.^11^ retrospectively reviewed 13 patients at 25–63 months post-surgery, assessing tendon healing, tendinopathy and fatty atrophy. Clinically and, particularly in the context of early post-operative re-injury requiring subsequent imaging, a better understanding of the MRI-based appearance of the repair throughout the post-operative timeline may provide clinicians with an idea of what to expect and better guide the requirement of further intervention, including re-operation.

The current study sought to develop a proximal hamstring repair MRI tool, further seeking to investigate the post-operative MRI-based improvement of the repair and its correlation with clinical outcome. It was hypothesized that: 1) the MRI-based scoring tool would demonstrate adequate intra- and inter-observer reliability, 2) the MRI-based appearance of the repair construct would significantly improve over time up until 12-months post-surgery, and 3) clinical and MRI-based outcomes would be highly correlated.

## 2 Materials and methods

Between November 2020 and June 2021, 15 patients (10 males, 67 %) underwent proximal hamstring tendon repair due to a complete 3-tendon rupture, confirmed pre-operatively via MRI and intra-operatively. Patients presented with a mean age of 51.4 years (range 34–65), body mass index (BMI) of 26.7 (range 21.3–38.3) and injury to surgery timeframe of 29.7 days (range 5–56). The operated limb was the patient's dominant side (preferred kicking limb) in 7 (47 %) patients. The reported injury mechanism was during sport/recreational activity in 9 (60 %) patients, and accidents at home or work in 6 (40 %) patients. Ethics approval was obtained by the relevant university human research ethics committee.

### 2.1 Operative technique

Briefly, all surgical repairs were undertaken with the patient positioned in prone. A transverse incision at the level of the buttock crease was generally employed (for acute avulsions and/or tendon injuries with <3 cm of retraction), though a vertical incision was considered if the injury was >3 weeks prior to surgery and/or retracted >3 cm on pre-operative MRI.

Superficial dissection was undertaken, initially identifying the gluteus maximus muscle, with the inferior gluteal neurovascular bundle identified and subsequently protected. The inferior gluteal maximus muscle margin was mobilized and retracted to maintain exposure. The sciatic nerve was identified and dissected, mobilized laterally, retracted and carefully protected.

The proximal hamstring tendon mass was then identified and a 5 Tycron suture was locked to the proximal free end, allowing tension to the free tendon end to dissect scar tissue and mobilize the tendon mass. The lateral, posterior and inferior wall of the ischium was debrided to bleeding bone to enhance tendon bone healing. Three suture anchors (CrossFT Suture Anchors, ConMed, Largo FL) were placed onto the lateral (n = 2) and distal (n = 1) wall of the ischium for the repair, with sutures placed into the tendon mass in a locking and sliding technique. Two knotless anchors (CrossFT Knotless Deep Thread Suture Anchors, ConMed, Largo FL) were placed on the posterior wall of the ischium, and a knotless row of sutures were tensioned and secured onto the posterior wall. The sciatic nerve was inspected, with wound lavage undertaken and closure by layers.

Post-operatively, patients were instructed to touch weight bear over the first 2 weeks with crutches, though weight bearing was permitted as tolerated after that early period. Patients were fitted for a hinge knee brace set at 30° to be worn for the first 2 weeks. Early activity was restricted, with swimming, cycling and closed kinetic chain and strengthening exercises introduced from 6 weeks post-surgery.

### 2.2 MRI protocol and hamstring scoring

The proximal hamstring repairs were evaluated using high resolution MRI pre-operatively and at 3-, 6- and 12-months post-surgery, using a Philips 3T Elition X scanner (Software version R5.7). Standardized T1 and T2 mDIXON images were obtained in the coronal plane (thickness 3.5 mm, FOV 350 mm FH and 370 mm RL, 576 x 576 matrix), with T1 and Proton Density-weighted fat-saturated (PDFS) images obtained in the axial plane (thickness 4 mm, FOV 190 AP and 360 RL, 1024 x 1024 matrix).

**Fig. 1:**
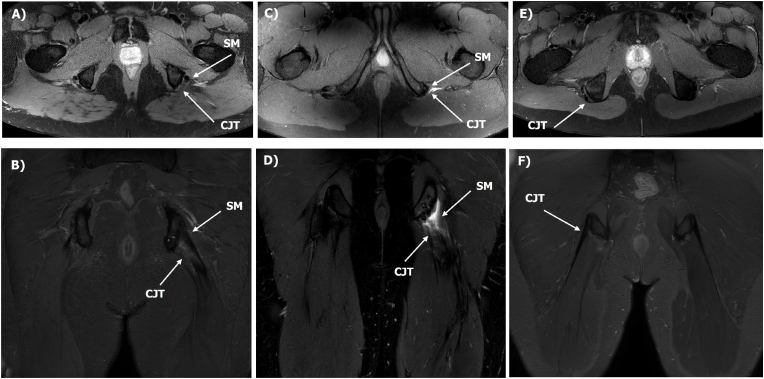
Proximal Hamstring Objective Magnetic Resonance Imaging Score (PHOMRIS) images representing: (A) axial and (B) coronal plane images of the semimembranosus (SM) scored at 1 and conjoint (CJT) scored at 7, (C) axial and (D) coronal plane images of the SM scored at 7 and CJT scored at 7, and (E) axial and (F) coronal plane images of the CJT scored at 1.

**Fig. 2:**
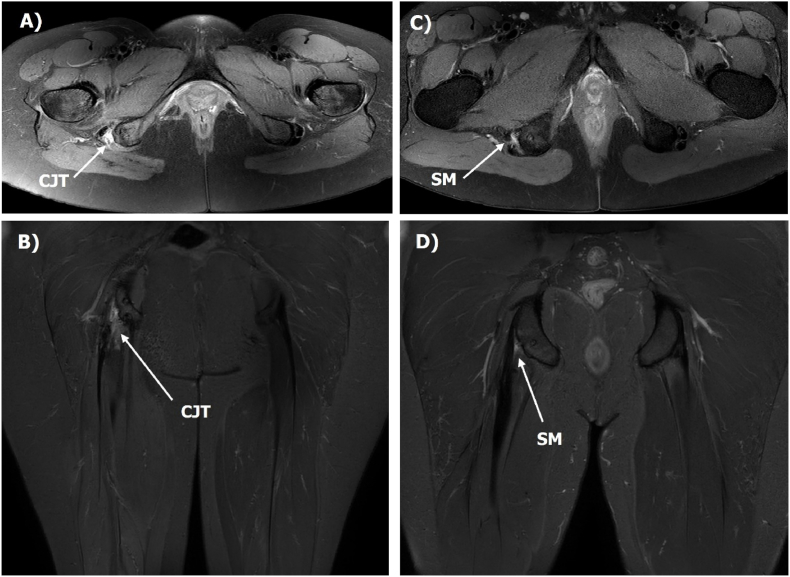
Proximal Hamstring Objective Magnetic Resonance Imaging Score (PHOMRIS) images representing: (A) axial and (B) coronal plane images of the conjoint (CJT) scored at 5, and (C) axial and (D) coronal plane images of the semimembranosus (SM) scored at 2.

An MRI-based scoring tool was then employed to score the hamstring repairs (Proximal Hamstring Objective Magnetic Resonance Imaging Score - PHOMRIS), originally drafted based on the senior author's experience and then further developed and finalized in consultation with other orthopaedic specialists, the radiology and academic/research teams. Both the conjoint (semitendinosus & biceps femoris) and semimembranosus components of the proximal insertion were each assessed according to this score, based on bone-tendon interface healing, signal and degree of displacement/retraction (Table 1). Examples of images and the respective PHOMRIS scores are show in Fig. 1, Fig. 2.

**Table 1:** The Proximal Hamstring Objective Magnetic Resonance Imaging Score (PHOMRIS) employed to evaluate the semimembranosus and conjoint (semitendinosus & biceps femoris) components of the proximal hamstring insertion (with a higher score indicating a greater degree of T2 high signal at the bone-tendon interface with a greater degree of tendon displacement).

Grade	Description
1	No high signal between hamstring and ischium
2	<50 % of bone-tendon interface has T2 high signal (≥50 % footprint healing)
3	≥50 % of bone-tendon interface has T2 high signal (<50 % footprint healing, no displacement)
4	100 % of bone-tendon interface has T2 high signal (no displacement)
5	100 % of bone-tendon interface has T2 high signal (<1 cm displacement)
6	100 % of bone-tendon interface has T2 high signal (1–2 cm displacement)
7	100 % of bone-tendon interface has T2 high signal (>2 cm displacement)

### 2.3 Clinical outcome measures

PROMs were completed pre-operatively and at 3-, 6- and 12-months post-operatively. Firstly, the PHAT was employed, scored from 0 to 100, assessing hamstring-associated pain (during sitting, striding out, when on stretch and at rest), hamstring/buttock tenderness, time permitted undertaking various activities without discomfort (sitting, driving, running) and current activity level.^12^ The LEFS, scored from 0 to 80, was employed to assess functional abilities.^13^^,^^14^ The Tegner Activity Scale (TAS)^15^ was also employed to evaluate current activity level and is scored from 0 (‘sick leave/disability’) to 10 (‘participation in competitive sports such as soccer at a national or international elite level’). At 6- and 12-months, a Global Rating of Change (GRC) scale was also employed evaluating the patient's perceived recovery. This was scored from −5 (much worse) to 0 (the same) to 5 (completely recovered).

### 2.4 Data and statistical analysis

Given the primary outcome of this study was to develop and formally apply the PHOMRIS tool in a patient population undergoing proximal hamstring tendon repair, a consecutive patient sample of n = 15 (with serial MRI at 3, 6 and 12 months post-surgery) was deemed appropriate. With regard to the MRI scoring system, inter- and intra-observer reliability was assessed using the Spearman's rank order (rho) correlation for each proximal hamstring component (semimembranosus and conjoint). Inter-observer reliability was assessed by two independent radiologists scoring 45 MRI studies (MRIs undertaken at 3-, 6- and 12-months post-surgery in all 15 patients). Intra-observer reliability was assessed by one of the radiologists re-scoring 15 randomly selected MRI studies, which was undertaken 2 weeks after the initial scoring. Means (SD and range) for all measures were calculated, while Analysis of Variance (ANOVA) evaluated change over the pre- and post-surgical timeline. Correlational analysis was used to assess the association between clinical (PROMs) and MRI-based scores. Statistical significance was determined at *p* < 0.05, with analysis performed using SPSS (SPSS, Version 27.0, SPSS Inc., USA).

## 3 Results

Inter-observer reliability indicated a strong correlation for the semimembranosus (rho = 0.827, p < 0.0001) and conjoint (rho = 0.851, p < 0.0001) tendon components. Intra-observer reliability indicated a strong correlation for the semimembranosus (rho = 0.852, p < 0.0001) and conjoint (rho = 0.996, p < 0.0001) tendon components.

All PROMs, as well as the radiological scoring for the semimembranosus hamstrings component improved (p < 0.05) (Table 2). No change over time was observed for the radiological scoring for the conjoint hamstrings component (p = 0.219) (Table 2). Several significant correlations (p < 0.05) were observed between PROMs at 12-months (Table 3). While a significant negative association was seen between the LEFS and the MRI-based score for the semimembranosus component (r = −0.57, p = 0.042), no other significant correlations (p > 0.05) were observed between clinical and MRI measures (Table 3).

**Table 2:** Patient-reported outcome measures (PROMs) and MRI-based scoring measures over the pre- and 12-month post-operative timeline.

Variable	Pre-surgery	3-months	6-months	12-months	p value
SM	5.5 (2.4), 1-7	3.9 (2.5), 1-7	3.9 (2.5), 1-7	3.8 (2.6), 1-7	0.039
CJ	5.8 (2.1), 1-7	4.6 (2.8), 1-7	4.6 (2.8), 1-7	4.6 (2.8), 1-7	0.219
PHAT	34.7 (17.5), 2-68	63.2 (16.3), 22-90	65.6 (12.4), 35-85	79.2 (13.5), 52-100	<0.0001
LEFS	33.2 (20.0), 0-65	56.9 (7.7), 42-74	64.4 (9.9), 47-79	71.5 (11.9), 40-80	<0.0001
TAS	2.9 (1.8), 0-6	3.4 (1.1), 2-6	4.0 (1.4), 3-7	4.7 (1.9), 2-9	0.026
GRC	N/A	N/A	2.1 (2.3), -4-4	2.9 (1.9), -1-5	N/A

SM = semimembranosus; CJ = conjoint; PHAT = Perth Hamstring Assessment Tool (PHAT); LEFS = Lower Extremity Functional Scale; TAS = Tegner Activity Scale; GRC = Global Rating of Change Scale.

**Table 3:** 12-month post-operative correlations undertaken between MRI-based scores and patient-reported outcome measures.

	SM	CJ	PHAT	LEFS	TAS	GRC
SM	1.00					
CJ	0.65^b^	1.00				
PHAT	−0.32	−0.20	1.00			
LEFS	−0.57^a^	−0.25	0.66^a^	1.00		
TAS	−0.46	−0.33	0.68^b^	0.65^a^	1.00	
GRC	−0.27	0.21	0.33	0.42	−0.11	1.00

SM = semimembranosus; CJ = conjoint; PHAT = Perth Hamstring Assessment Tool (PHAT); LEFS = Lower Extremity Functional Scale; TAS = Tegner Activity Scale; GRC = Global Rating of Change Scale.

a Correlation is significant at the 0.05 level (2-tailed).

b Correlation is significant at the 0.01 level (2-tailed).

## 4 Discussion

The current study demonstrated excellent reliability of the proximal hamstring repair MRI tool (PHOMRIS) developed, based on factors such as bone-tendon interface healing, signal and retraction. Such a tool may be used in both a research and clinical setting, with the 3-, 6- and 12-month MRIs undertaken in the cohort of patients included in the current study also providing the specialist with a better understanding of the MRI-based appearance of the post-operative repair in the context of early post-operative re-injury requiring subsequent imaging throughout the post-operative timeline.

PHOMRIS demonstrated excellent inter- and intra-observer reliability, in support of the first study hypothesis. Limited published evidence exists on the MRI-based appearance of the proximal hamstring repair construct, let alone a scoring tool that may be employed to assess the repair status over time. At 6 months, Lefevre et al.^16^ reported a healed hamstring tendon on MRI in their series of patients undergoing surgery for acute tears, also reporting no observed fatty muscle degeneration or atrophy. Mica et al.^10^ retrospectively reviewed the MRIs of six patients at 10–118 months following proximal hamstrings repair, reporting complete consolidation in all patients, without evidence of fatty muscular degeneration. Chahal et al.^11^ retrospectively reviewed the MRIs of 13 patients at 24–62 months post-surgery, with all patients demonstrating a healed repair and evidence of tendinopathy and mild atrophy in 25 % of patients. Where reported, only a single radiologist was enlisted in each of the aforementioned studies to review the MRIs.

A range of systematic reviews have been published presenting outcomes of patients undergoing proximal hamstring repair,^4^, ^5^, ^6^, ^7^, ^8^ generally demonstrating good functional outcomes, strength restoration and high satisfaction and RTS rates, with better outcomes in acute (versus chronic) repairs and better functional outcome and strength restoration in patients undergoing repair for partial (versus complete) tears. Subsequent to the MRI analysis, PROMs significantly improved over the 12-month post-operative period. With respect to the PHAT and LEFS, recommended for use in the post-operative patient assessment of proximal hamstring repair,^9^ the 12-month mean scores reported were similar to those reported over the past 10 years in the context of acute repair,^2^^,^^11^^,^^17^, ^18^, ^19^ as was the case with the TAS.^11^^,^^16^^,^^20^^,^^21^

Using the developed MRI-based scoring tool, the current study sought to evaluate the appearance of the repair construct periodically over the first 12 post-operative months in a consecutive series of patients undergoing surgery, and assess the correlation between clinical and MRI-based outcome at 12 months. While both the semimembranosus and conjoint components improved from pre-to post-surgery, this was not statistically significant for the conjoint tendon attachment, only partially supporting the second hypothesis. Despite the improvement in the semimembranosus component, the lack of statistically significant improvement in MRI-based healing of the conjoint tendon component over the 12-month post-operative period was an unexpected finding. One possible explanation is the observation of the conjoint tendon attachment being partly muscle and less tendinous at the distal attachment onto the ischium. Surgical repair and healing may therefore be limited, promoting the scores observed. Furthermore, the end-to-end attachment of the conjoint tendon to the distal ischium may not offer the stronger repair observed via the side-side repair alignment of the semimembranosus to the lateral ischium wall. Nonetheless the improvement observed on MRI in both tendon components was observed largely within the first 3 months, with little (if any) further improvement from 3 to 12 months post-operatively.

Subsequently, a relative lack of correlation was observed between these clinical and MRI-based outcomes, with the third hypothesis also only partially supported. Of interest, while the LEFS was significantly correlated with the semimembranosus hamstring component at 12 months, patient-reported activity level (TAS) as well as the PHAT which is a more specific proximal hamstring assessment PROM, were not. While not reported previously after proximal hamstring repair, a lack of correlation between post-operative clinical and MRI-based outcomes has been observed after other tendon-related surgical procedures. After hip abductor tendon repair, McGonagle et al.^22^ reported that the MRI appearance of the tendon did not normalize after surgery and there was limited correlation between post-operative MRI appearances (pre- or post-surgery) and clinical outcome. Similarly, after rotator cuff repair surgery, a review published by Lee et al.^23^ highlighted the clinically insignificant abnormal imaging appearances observed after repair. Crim et al.^24^ reported large variability in tendon appearance after surgery, with improved appearance after surgery (up until 12 months) though no correlation between MRI-based appearance and function. More recently, Paul et al.^25^ reported a dissociation between MRI-based cuff healing and post-operative shoulder function. A meta-analysis had also previously reported no clinically relevant difference in pain or function based on structural integrity after cuff repair.^26^

A range of study limitations are acknowledged. No priori sample size calculation was performed, rather a consecutive series of 15 patients with post-operative MRIs undertaken at 3-, 6- and 12-months was selected prior to study onset given the nature and primary aim of the study (development and reliability assessment of the MRI-based assessment tool), pilot use of the MRI-based scoring tool and cost associated with non-clinical post-operative serial MR imaging. With a larger cohort, the significance of the correlations investigated may change, albeit this was not the primary outcome of the current study. Secondly, while use of the tool may be broad across clinical and research settings, as well as when employing varied surgical techniques, the clinical and MRI-based outcomes of the cohort presented may be limited to the specific surgical technique employed. Thirdly, we employed the PHAT and LEFS which have been recommended for use in the post-operative patient assessment of proximal hamstring repair.^9^ However, other published studies reporting outcomes after proximal hamstring repair have employed other PROMs including the Proximal Hamstring Injury Questionnaire (PHIQ),^27^ Single Assessment Numeric Evaluation (SANE)^28^ and University of California at Los Angeles (UCLA) 10-point scale,^16^ as well as other hip-specific PROMs such as the 12-item International Hip Outcome Tool (iHOT-12),^20^ the Kerlan-Jobe Orthopaedic Clinic (KJOC) Athletic Hip score^20^ and the Harris Hip Score.^21^ Further to this, we employed the TAS to assess activity which has been used by others,^11^^,^^16^^,^^20^^,^^21^ though the Marx has also been reported.^2^^,^^17^^,^^20^^,^^29^

## 5 Conclusions

The current study sought to develop and assess a proximal hamstring repair MRI tool (PHOMRIS), which demonstrated excellent inter- and intra-observer reliability. Such an MRI-based scoring tool may provide benefit in the context of research, while serial post-operative MRI will provide insight to the clinician of what to expect in the clinical context of early re-injury that may warrant further imaging, with subsequent consideration for re-operation. Of interest, most of the MRI-based improvement appeared to occur within the first 3 months, with little further change following that time.

## Funding/financial disclosure

Independent funding in the form of a research grant was provided by ConMed Corporation to assist this research.

## Author contributions

The following authors have conceived and designed the study (JE; WB; SK; PA), supervised the conduct of the study (JE; WB; SK; PA), analyzed the data (JE; WB; SK), wrote the initial drafts (JE), critically revised the manuscript (JE; WB; SK; PA) and ensure the accuracy of the data and analysis (JE; WB; SK; PA). I confirm that all authors have seen and agree with the contents of the manuscript and agree that work has not been submitted or published elsewhere in whole or part.

## Ethics approval

Ethics approval was obtained by the University of Western Australia (RA/4/20/4824) Human Research Ethics Committee (HREC).

## Consent to participate

Informed and written consent was obtained from all individual participants included in the study.

## Declaration of competing interest

We wish to confirm that there are no known conflicts of interest associated with this publication, though independent funding in the form of a research grant was provided by ConMed Corporation to assist this research.

We confirm that the manuscript has been read and approved by all named authors (as stated in our Cover Letter) and that there are no other persons who satisfied the criteria for authorship but are not listed.

We further confirm that the order of authors listed in the manuscript has been approved by all of us.

We confirm that we have given due consideration to the protection of intellectual property associated with this work and that there are no impediments to publication, including the timing of publication, with respect to intellectual property. In so doing we confirm that we have followed the regulations of our institutions concerning intellectual property. We further confirm that any aspect of the work covered in this manuscript that has involved either experimental animals or human patients has been conducted with the ethical approval of all relevant bodies and that such approvals are acknowledged within the manuscript.

We understand that the Corresponding Author is the sole contact for the Editorial process (including Editorial Manager and direct communications with the office). He is responsible for communicating with the other authors about progress, submissions of revisions and final approval of proofs. We confirm that we have provided a current, correct email address which is accessible by the Corresponding Author and which has been configured to accept email from jay.ebert@uwa.edu.au.
